# Management of Coexistence and Conflicts Between Humans and Macaques in Japan

**DOI:** 10.3390/ani15060888

**Published:** 2025-03-20

**Authors:** Léane Depret, Cédric Sueur

**Affiliations:** 1 ANTHROPO-LAB, ETHICS EA 7446, Université Catholique de Lille, 59000 Lille, France; leane.depret.etu@univ-lille.fr; 2 Universite de Strasbourg, IPHC, CNRS, UMR 7178, 67087 Strasbourg, France; 3 Institut Universitaire de France, 75231 Paris, France

**Keywords:** conflicts, primates, urbanisation, coexistence, anthrozoology, behavioural ecology, rewilding, Japan

## Abstract

Conflicts between humans and Japanese macaques (*Macaca fuscata*) have intensified due to urban and agricultural expansion, reducing natural habitats and pushing macaques into human settlements. This review examines strategies for managing human–macaque coexistence in Japan. Since the 1970s, urbanisation and deforestation have led to increased macaque migration into populated areas, resulting in property damage, crop loss, and, in some cases, aggressive encounters. The growing macaque population underscores the urgency of effective management programmes. Strategies include preventive measures, such as weeding to limit food sources, habitat modification, and community-based interventions to reduce attractants. Non-lethal deterrents, including visual and auditory scare tactics, trained guard animals, and electric fences, have been implemented to discourage macaques from entering human spaces. Physical barriers, such as fences and buffer zones, provide long-term mitigation but require maintenance and community cooperation. Fertility control, including sterilisation and hormonal contraception, offers a long-term population management solution but presents logistical challenges. Lethal control and capture-relocation, though controversial, remain options for particularly problematic individuals. Additionally, integrating One Health and One Conservation approaches into macaque management allows for a holistic strategy that considers disease risks, ecological balance, and ethical implications. A balanced management plan that incorporates multiple strategies, community participation, and continuous monitoring is crucial for mitigating conflicts and fostering sustainable coexistence between humans and macaques.

## Simple Summary

Conflicts between humans and Japanese macaques (*Macaca fuscata*) have become more frequent, due to both urban and macaques’ expansions which push these primates into human settlements. This review explores strategies used in Japan to manage these conflicts, balancing conservation with reducing negative interactions. Measures include habitat modification, deterrence techniques, and fencing to prevent macaques from accessing human areas. In severe cases, controlled capture–either relocation or lethal removal—is used. This paper evaluates the effectiveness of these strategies, highlighting the importance of adopting approaches to different levels of conflict. Sustainable coexistence requires an integrated approach that considers ecological, social, and cultural factors. This research provides valuable insights for policymakers and communities seeking humane and effective solutions for managing human–wildlife conflicts worldwide.

## 1. Introduction

As human populations expand into areas previously untouched by development, interactions between humans and wildlife are becoming increasingly frequent and conflictual [1]. These conflicts arise from various causes, including competition for natural resources, habitat destruction, and predation by wild animals on domestic livestock [2,3,4,5]. Cultural and ontological differences–shaped by varying conceptions of reality and the relationship between humans and nature–also influence how societies perceive and manage these conflicts, leading to diverse approaches tailored to specific contexts [6,7,8].

Urban expansion is a significant factor driving these conflicts, as it fragments natural habitats, restricts the dispersal of animal populations, and reduces opportunities for mate finding and gene flow [9,10]. Wild animals often become isolated in habitat fragments surrounded by urban or rural areas, limiting their access to essential resources like food and water. As a result, they are forced to venture into human-inhabited zones, leading to encounters that can cause livestock predation, crop destruction, and property damage [11,12,13]. Conversely, urban or anthropogenic environments can provide easier access to high-calorie food sources and lower mortality risks from hunting or tracking, further encouraging wildlife to exploit these areas.

Farmers, in particular, bear substantial economic losses due to wildlife-induced damage to crops and livestock. Efforts to mitigate these conflicts include awareness campaigns, the adoption of sustainable agricultural practices, the construction of physical barriers, and the implementation of non-lethal deterrent techniques [1,5,7,14,15,16,17]. However, it is crucial to acknowledge that each conflict scenario is unique, necessitating context-specific strategies to develop effective and equitable solutions for all stakeholders [16].

The Japanese macaque (*Macaca fuscata*) is a species frequently involved in conflicts with local populations, prompting the development of various solutions tailored to the level of conflict [13,18,19,20,21,22,23,24]. Existing publications addressing measures to mitigate crop damage caused by Japanese macaques are often written in Japanese or provide only brief, non-exhaustive descriptions of available methods.

This study aims to systematically illustrate, through a scoring framework, the range of control measures implemented by the Japanese government and the Shiga Prefecture. Shiga Prefecture, located in central Japan and home to Lake Biwa, Japan’s largest freshwater lake, is a region where urban expansion, agriculture, and forested landscapes intersect, making it a key area for studying human–macaque conflicts and the implementation of diverse management strategies. These measures span from non-lethal to lethal approaches, depending on the severity of the damage caused by macaques. The analysis is based primarily on the translation and interpretation of government reports [25] and Shiga Prefecture reports [26] on Japanese macaque population management. This work represents an analysis of existing literature rather than insights gained from direct field observations. Notably, these reports had remained untranslated into English or French, and their methodologies had not been disseminated in academic literature beyond the Japanese language until the present study.

Following an overview of the historical context and the damage caused by Japanese macaques, this study will detail the various strategies employed to manage conflicts between humans and macaques, providing a comprehensive perspective on existing management practices.

## 2. Historical Background of the Japanese Macaque–Human Relationship

The Japanese macaque (Macaca fuscata) [27], commonly known as the snow monkey, is a medium-sized primate native to Japan. Males typically weigh between 10 and 14 kg, with some individuals reaching up to 18 kg, while females are significantly smaller, weighing around 5 to 9 kg. Their body length ranges from 47 to 60 cm for males and 45 to 55 cm for females, with a relatively short tail measuring approximately 8 to 10 cm. Japanese macaques exhibit strong sexual dimorphism, with males being both larger and more dominant within their social hierarchy.

Japanese macaques live in multi-male, multi-female social groups, which vary in size from 20 to over 100 individuals, depending on habitat quality and resource availability [28,29]. These groups are structured around female matrilines, meaning that females remain in their natal groups for life, forming stable kinship networks, while males disperse upon reaching maturity. Within the group, a dominance hierarchy exists among both males and females, influencing access to resources and mating opportunities. Social cohesion is maintained through grooming, vocal communication, and cooperative behaviours, which also help to mitigate conflicts within the group.

**Figure 1:**
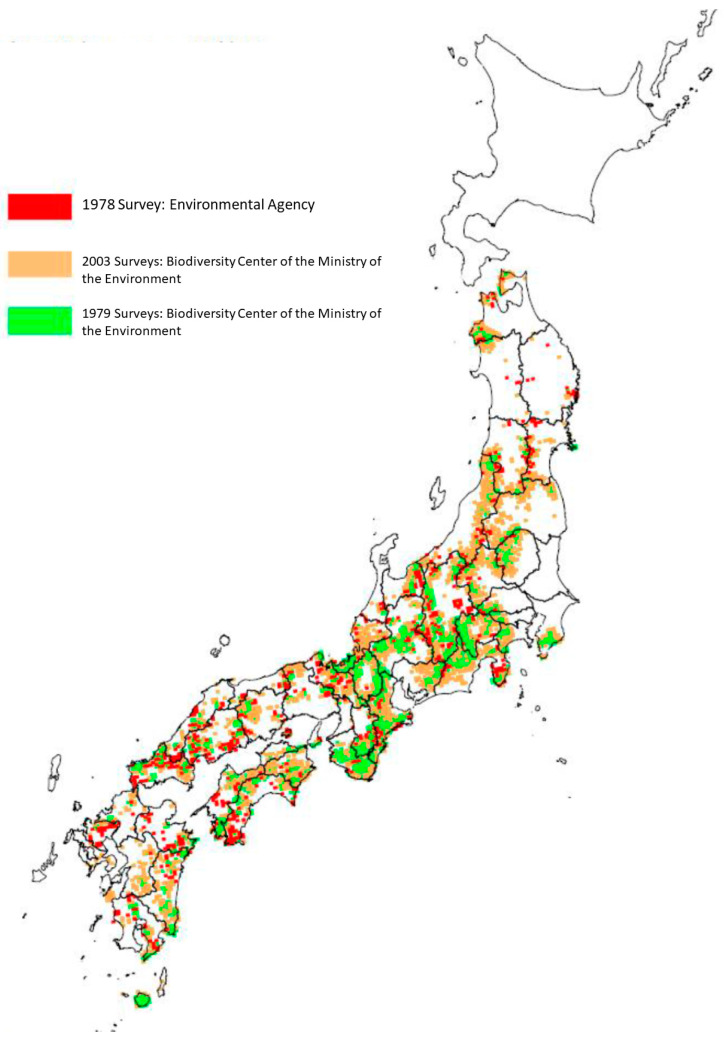
Distribution of Japanese macaque troops based on an internal Japanese document provided by Kunio Watanabe, a primatologist professor at Kyoto University and head of the Japanese macaque management group (1978 Survey: Environmental Agency; 1979 and 2003 Surveys: Biodiversity Center of the Ministry of the Environment, 2004). The status of the Japanese macaque habitat shows a significant increase in their distribution across Japan. National surveys on their distribution were conducted in 1978 and 2003. Macaque troops are present in 43 prefectures, except for Hokkaido, Ibaraki, Nagasaki, and Okinawa. Over the past 25 years, their range has expanded by approximately 1.5 times. The most significant expansions were observed in the Tohoku and Kanto regions, where distribution increased by 2.3 times and 1.9 times, respectively. Other regions, such as Chugoku, experienced a smaller expansion at 1.02 times, while Chubu increased by 1.5 times, and Kinki and Shikoku by 1.4 times each. The Kyushu region showed an increase of 1.6 times.

Japanese macaques are an endemic monkey species found across various ecosystems in Japan [27]. Their distribution spans from the humid subtropical forests of Yakushima in the south to the subarctic mountainous regions such as Jigokudani in the north (Figure 1). These semi-terrestrial primates spend most of their time on the ground but sleep in trees. They are omnivorous, feeding primarily on plants and fruits, but their diet also includes insects, bark, fish, fungi, and even soil [28,30,31,32,33,34].

Japanese macaques arrived on the Japanese archipelago via the Korean Peninsula approximately 430,000 years ago. Before the 12th century, their distribution was largely distant from human settlements [35]. During this period, sparse forests were uncommon, and the population density of macaques was relatively low. The Japanese archipelago was predominantly covered by dense deciduous and coniferous forests, providing abundant natural habitats that supported a rich diversity of wildlife. Human activity was primarily centred around hunting and fishing for subsistence, which did not significantly disrupt wildlife [36,37].

The low population density of both macaques and humans, combined with the macaques’ preference for dense and closed habitats, kept them far from human habitation. These forests offered macaques protection from predators and supported their small group sizes, encouraging them to remain in isolated environments [35,38,39,40]. The densely mixed deciduous and coniferous forests were crucial for their survival and behavioural ecology, providing the cover necessary to avoid predation and thrive in their natural environment.

There is limited information about the lifestyle of Japanese macaques during the Middle Ages (12th to 16th centuries). At that time, Japanese forests played a central role in daily life and the economy, with their use carefully managed to balance human needs and the preservation of forest ecosystems. However, this balance shifted over time due to social and economic changes [41,42]. According to Hirose [43], museums do not sufficiently represent the role of monkeys in Japanese spirituality, showcasing primarily positive artefacts such as origami, ema (painted wooden tablets), and kukurizaru (ball talismans symbolising monkeys). In contrast, Japanese macaques were often depicted satirically in 14th-century art [44].

During the Edo period (1603–1868), much of Japan was characterised by sparse pine forests, grassy hills, and barren mountains–a result of prolonged timber exploitation during earlier military conflicts, particularly at the onset of Tokugawa rule. The Edo period saw the implementation of forest conservation and management policies aimed at balancing resource use with preservation. Despite these efforts, increased urbanisation later in the period led to significant changes in forest landscapes [45,46,47]. Wildlife management during this time aimed to balance human needs with environmental conservation [37]. Hunting of Japanese macaques was banned in western Japan, but this restriction had minimal impact on communities and farmland since macaques were seldom mentioned in written records. The primary game species during this time were sika deer and wild boar, as hunting generally occurred near villages rather than in the dense forests where macaques resided [35]. These forests, which were far from human settlements, provided macaques with refuge, making them less likely to be hunted. However, as human populations grew and settlements expanded deeper into mountainous regions, conflicts with wildlife became more frequent. There are a few documented cases of damage caused by Japanese macaques during the Edo period. For example, in the Nagai region of Niigata Prefecture and the Tohoku region, farmers had to vigilantly protect their crops, particularly in mountainous areas where macaques encroached on agricultural land [35].

During the Meiji period (1868–1912), Japan’s forests and their relationship with society underwent profound changes due to modernisation, industrialisation, and socio-economic reforms [48,49,50]. The increasing demand for forest resources, exacerbated by the environmental devastation caused by the Sino–Japanese and Russo–Japanese Wars, led to unprecedented levels of deforestation. This period is regarded as one of the most destructive for Japan’s forests, with long-lasting consequences for wildlife. The survival environment for Japanese macaques became severely restricted.

The Oil Revolution of the 1950s further diminished the role of forests in daily life. Forests were no longer relied upon for firewood, fertiliser, or food for domestic animals. Additionally, the importation of foreign timber made domestic forest resources less critical for construction, leading to the abandonment of forests for industrial purposes in Japan [51,52]. Rural populations declined as people migrated to urban areas, allowing natural vegetation to reclaim former agricultural lands, even near villages and cities. This regrowth created favourable conditions for wildlife, including Japanese macaques, to expand into new territories. The extinction of the Japanese wolf [53,54,55], a natural predator of macaques, further facilitated the rapid expansion of macaque populations into new habitats.

In the 1950s, the Japanese macaque population was critically small, with only a few dozen individuals captured annually due to crop damage. Factors such as hunting, predation, and the destruction of forest habitats severely limited their distribution during this time. However, by the 1960s, the population began to recover significantly. By 1978, over 2000 macaques were being captured and killed annually as part of conflict mitigation efforts [35]. Urbanisation continued to expand, encroaching on natural habitats and increasing the frequency of human–wildlife interactions.

Wildlife management became a pressing concern to address conflicts between humans and Japanese macaques while ensuring the protection of wildlife. One effective approach to managing these interactions is ‘adaptive management’ [6,56]. Adaptive management is a flexible and dynamic framework that incorporates continuous monitoring, regular evaluations, and iterative adjustments based on observed outcomes. It recognises that environmental conditions, animal behaviours, and human needs are constantly changing, requiring strategies to evolve accordingly. This approach allows for the development of balanced and sustainable solutions to mitigate human–macaque conflicts, while accounting for the complexity and variability of ecological and social systems.

The 1970s marked a period of significant transformation for Japanese wildlife, characterised by the rapid expansion of populations of Japanese macaques, sika deer, and wild boars [37]. Factors that previously limited their distribution, such as hunting, predation, and habitat destruction, had largely disappeared. Meanwhile, urban expansion following World War II fragmented and threatened natural habitats, particularly forests, intensifying efforts to protect the remaining natural areas. These concerns became central to environmental movements in the 1970s and 1980s, which raised awareness and led to the adoption of policies promoting sustainable forest management and ecosystem conservation. Despite these advances, challenges remain, requiring innovative strategies to integrate environmental protection into urban and rural development while preserving biodiversity and essential ecosystem services [23,57,58]. Wildlife management measures were implemented to promote harmonious coexistence between humans and wildlife [59,60]. However, the challenges of coexistence have only grown. By the early 2010s, there were an estimated 3000 groups of Japanese macaques, totalling approximately 180,000–200,000 individuals—a significant population increase in less than a century [61,62]. Lethal capture of Japanese macaques also rose sharply during this period, exceeding 20,000 annually in the 2010s and reaching 30,000 in recent years [35].

Coexistence between humans and Japanese macaques has become a significant challenge. These intelligent animals, with high learning capacities, often adapt to human environments and can become aggressive, particularly when seeking food. This issue is not unique to macaques; the Japanese press and scientists have also reported increased interactions with wild boars and bears near farms and urban areas [63,64]. These conflicts were driven by easier access to high-quality food sources in human environments and the potential impacts of climate change on natural resources [65]. Addressing these issues requires comprehensive management strategies that account for ecological, social, and environmental factors to ensure sustainable coexistence.

## 3. Current Damage Estimates from Japanese Macaques

**Figure 2:**
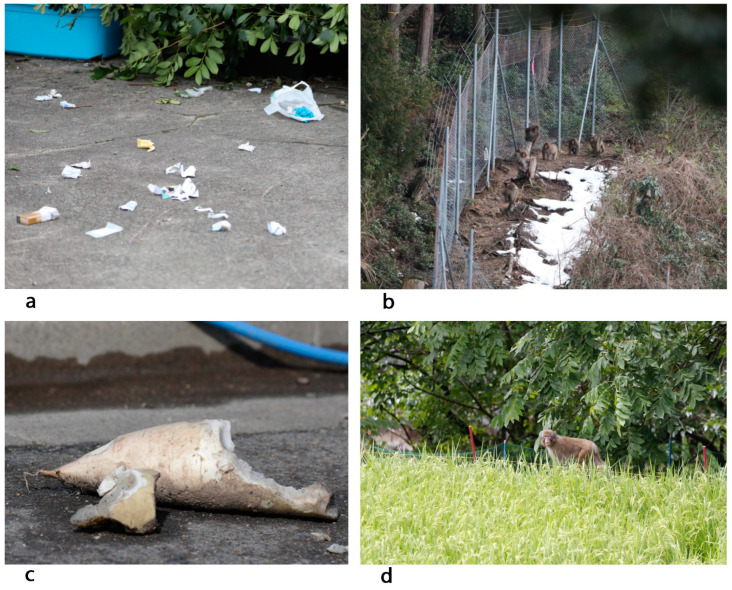
Damage caused by Japanese macaques (**a**,**c**), and installation of a fence around a forest (**b**) and a rice field (**d**). Photo credit: Cédric Sueur.

The presence of Japanese macaques in inhabited areas creates numerous challenges, particularly regarding the safety of residents and the protection of cultivated land [19,24,66]. Damage caused by macaques is increasing in human environments, including property destruction and intrusions (Figure 2a), as well as personal injuries reported in various regions [23,25,26]. Notably, this was observed in Yamaguchi in 2022, raising growing concerns among residents about the escalating impact of macaques.

The economic burden is significant, with Japanese macaques causing an estimated annual cost of 320,000 JPY per hectare [19]. In response, some farmers resort to legal or illegal killings, which can exacerbate the problem or escalate conflicts [15,23,67,68,69,70]. For example, non-selective culling often results in the formation of smaller, harder-to-track groups, which can cause even more widespread and unpredictable damage [24].

Despite protective measures, the number of macaques entering human settlements continues to rise, leaving many regions struggling to manage the issue. Furthermore, the macaques’ diurnal behaviour creates significant psychological stress for residents, who frequently witness the destruction caused by these animals. As captured in comments by Professor Kunio Watanabe in Cédric Sueur’s 2023 film Saru, a Story of Cultural Transmission [71], Watanabe observed the following:

‘I began my research in primatology in the 1970s. Back then, as soon as macaques saw us, they would run away. They were practically invisible, no matter where they were. This is a new generation we are dealing with. I’m now over 70 years old, and I’ve observed the changes in their behaviour. It’s undeniable. Today, we see monkeys in the middle of cities like Tokyo, Kyoto, and Osaka.’

Residents of Konan village in Shiga Prefecture describe similar issues in this movie:

‘Now they eat the bulbs too, don’t they?’; ‘Yes, they eat them too! I’ve even seen my tulip bulbs pulled out of the ground by the macaques. Last time, there were about thirty of them. Yes, even fifty.’; ‘We’d like to send them back to the mountains so they stop coming here. There are just too many of them.’; ‘It’s a nuisance–our crops are destroyed.’.

The term ‘nuisance’ refers to human–macaque conflicts that result in negative impacts on human activities, property, agriculture, or well-being. Typical nuisance behaviours in Japanese macaques are as follows:

- Crop Raiding: Damage to agricultural fields, orchards, and gardens, leading to economic losses for farmers.
- Property Damage: Macaques entering homes, damaging roofs, tearing screens, or rummaging through garbage.
- Aggressive Encounters: Incidents where macaques threaten or attack humans, particularly in areas where they have become habituated to human presence.
- Urban Intrusion: Macaques moving into residential areas, leading to concerns over safety and hygiene.

These testimonials highlight the growing frustration and urgency for effective management strategies to address the increasing conflict between humans and macaques.

**Figure 3:**
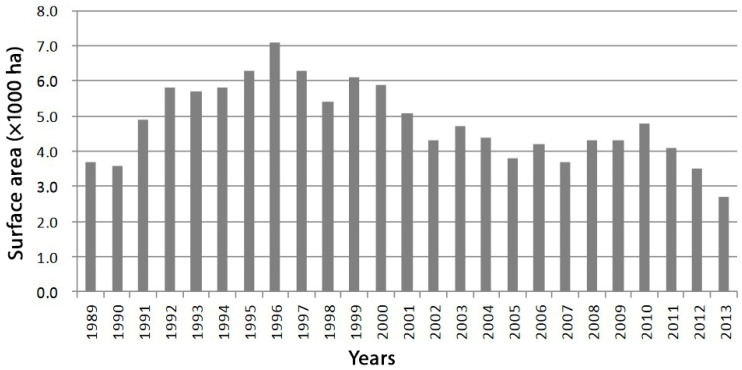
Evolution of the area of crop damage caused by Japanese macaques (in thousands of hectares). Modified figure from the Japanese Ministry of the Environment (Depret et al., 2023 [25]).

**Figure 4:**
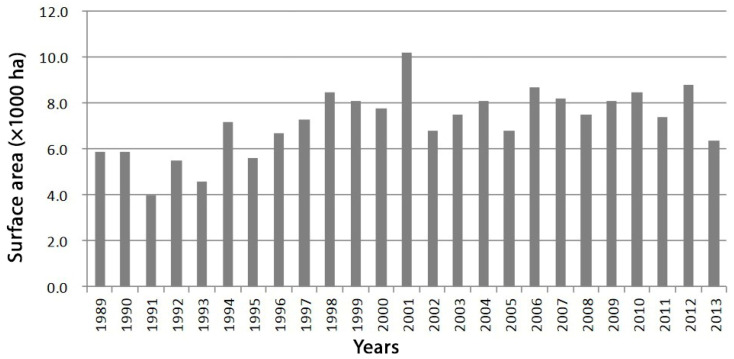
Evolution of the amount of crop damage caused by Japanese macaques (in thousands of tons). Created using data from the website of the Japanese Ministry of Agriculture, Forestry, and Fisheries.

Crop damage caused by Japanese macaques remains one of the most significant challenges in managing human–wildlife interactions. This damage primarily affects vegetable (Figure 2c) and rice (Figure 2d) crops. Since 2010, there has been a downward trend in the area impacted by macaque-related crop damage (Figure 3), and the overall volume of damage has stabilised (Figure 4). This improvement is largely due to the installation of preventive fencing and the implementation of comprehensive control measures, including both lethal and non-lethal capture, as well as effective deterrence techniques. However, certain regions, particularly mountainous areas, continue to experience significant crop losses. Intrusions, looting, and destruction are still commonly reported in villages such as Konan (from where the photos in Figure 2 were taken), Yudanaka (Nagano Prefecture), Tsumago (Gunma Prefecture), Yakushima Island, Shodoshima, and others (personal observations).

Reports from the Japanese government [25] and Shiga Prefecture [26] emphasise that the extent and type of damage caused by Japanese macaques vary significantly between groups. The severity and nature of damage are group-dependent; some groups cause little to no harm or have their impact mitigated through deterrent measures, while others inflict substantial damage despite protective efforts. For instance, a large troop of hundreds of macaques moving in unison can cause extensive destruction in a short period, whereas smaller groups tend to cause more localised and dispersed damage, which is harder to track and manage [24].

The seasonal movement patterns of Japanese macaques, driven by food availability [20,32], further complicate management efforts. Damage locations are not fixed and can shift throughout the year, making it challenging to predict and prevent incursions effectively.

## 4. Managing Cohabitation with Japanese Macaques

According to the macaque management group led by Kunio Watanabe and Hiroto Enari, in collaboration with the Japanese Ministry of the Environment, assessing the level of nuisance caused by Japanese macaques requires evaluating the specific degree of damage inflicted by each group rather than applying a one-size-fits-all strategy. This assessment is critical for determining the appropriate management strategies, such as capture methods and damage prevention measures. It also serves as a key indicator for evaluating the effectiveness of the measures implemented.

The Japanese Ministry of the Environment, in its Guide for the Development of the Specific Wildlife Protection and Management Plan [25], provides a framework to assess damage levels and the effectiveness of mitigation methods (Table 1). Various techniques, including field surveys, questionnaires, and expert evaluations, are used to analyse factors such as the frequency of group appearances, the average size of groups, their reactions to humans, and the degree of damage they cause to villages and daily life. Departments strive to locate and identify each macaque group and often track them using radio telemetry by equipping one individual from the group with a radio transmitter [24,25].

This system uses a scoring method to assign points to each indicator, with the total score compared to a reference table (Table 2) to classify the level of damage on a scale from 0 to 5. This classification facilitates the selection of appropriate prevention measures based on the specific behaviour and impact of the macaque groups.

In addition to the national framework, local governments may implement their own classification systems. For example, Shiga Prefecture evaluates damage levels using three indicators: frequency of occurrences, frequency of damage incidents, and macaque behaviour. These indicators are categorised into 10 levels, ranging from 1 to 10, allowing for a more nuanced approach tailored to regional needs. This flexibility in classification systems enables for a more effective management of human–macaque interactions while addressing the unique challenges posed by each group.

Damage prevention measures for Japanese macaques vary from town to town and include a range of strategies tailored to local needs. Common actions involve improved waste management, installation of fencing, and weeding to reduce macaque food sources. Scaring techniques, chasing animals away from sensitive areas, and creating buffer zones between macaque habitats and human settlements are also widely implemented. These measures aim to minimise interactions between macaques and humans. In addition, many areas install fencing to prevent macaques from intruding into crops and homes.

Capture operations, which may involve relocating or euthanising problem individuals, are often used to manage groups or individuals causing significant damage. This approach is not solely focused on population regulation but also on implementing measures to enable coexistence. However, descriptions of such cases in the literature are limited (for examples, see [72]). One notable case study by Morimitsu [73] in Kami, Hyogo Prefecture, details the simultaneous implementation of three countermeasures by the local government:

- Establishing an anti-monkey patrol group;
- Expanding the use of affordable and easy-to-install electric fences;
- Selectively capturing problem animals.

These efforts significantly reduced monkey sightings near fields (from 319 in 2010 to less than 100 in 2017) and agricultural damage (from 2,925,000 JPY in 2011 to 876,000 JPY in 2017). Under the guidance of the Working Group on the Conservation and Management of Japanese Macaques, chaired initially by primatologist Kunio Watanabe and now led by Hiroto Enari, management strategies have been strengthened through targeted captures of problematic individuals and groups. For long-term success, these measures must integrate damage prevention strategies to ensure balanced human–macaque coexistence. Considering both the number of groups and severity of impact (Table 1 and Table 2) allows for more effective planned capture operations and preventive measures, tailoring strategies to specific conflict levels.

Table 3 represents the strategy diagram used by the Shiga department [26]. Notably, Shiga Prefecture does not include sterilisation in its programme, despite scientific recommendations suggesting this technique as a way to manage local population abundance [61]. Such strategies aim to mitigate damage caused by Japanese macaques while preserving their populations and natural habitats. As the level of nuisance increases, the strategies become progressively more sophisticated and, in some cases, severe.

When preventive efforts fail to reduce damage, selective capture of the offending group can be employed to decrease its population. In extreme cases, total capture of a group is considered, particularly for groups that are entirely dependent on crops (e.g., those with an occurrence frequency level of 10 and damage level of 9 or higher, according to Shiga Prefecture standards). Captured macaques are usually relocated to remote areas, which may include the regions of their ancestors prior to their migration to human settlements, as well as islands or forests far from human activity. According to personal communication from Kunio Watanabe in 2023, these regions of origin are identified through demographic and genetic monitoring of macaque populations.

The Japanese generally avoid lethal measures, preferring relocation to co-exist peacefully with macaques. However, if individuals are deemed too aggressive and pose a threat to human safety, they are euthanised. In such cases, humane methods are used to ensure the animal is not subjected to unnecessary suffering, and carcasses are not used for experimental purposes, as the objective is population protection, not experimentation.

When carcasses are available, they are documented to support research on macaque management and conservation. Genetic and morphological analyses are conducted to study population variation and maintain genetic diversity among local populations [62,74,75]. Carcasses are disposed of properly, often through incineration or other methods, to prevent leaving remains in wild areas, which could attract scavengers or cause contamination.

To effectively protect and manage both Japanese macaques and human communities, an integrated approach is essential. This approach should combine three key components:

- Population regulation: Ensuring balanced macaque populations through targeted measures such as selective captures or sterilisation.
- Damage prevention: Implementing fencing, deterrents, and other measures to reduce macaque impact on crops and property.
- Habitat management: Preserving and managing the natural environment to reduce the likelihood of macaques encroaching on human areas.

These aspects must be applied in a way that reflects local conditions, ensuring a balanced and sustainable coexistence between humans and Japanese macaques.

Sterilisation has been explored as a non-lethal method for managing Japanese macaque populations, with varying levels of success and associated challenges. Early studies tested intrauterine contraceptive devices (IUDs) in female macaques, revealing prolonged mating seasons in non-pregnant females, while the presence of infants inhibited this effect [76]. Later research demonstrated that a progesterone-releasing intrauterine system effectively prevented conception without negatively impacting general health, behaviour, or menstrual cycle regularity [77]. Similarly, the use of copper intrauterine devices showed no adverse effects on ovarian, hepatic, or renal functions, though minor inflammatory responses were observed in endometrial tissues [78]. Male sterilisation, particularly castration, has also been considered as a management tool. While castrated males exhibited lower testosterone levels and reduced aggression, their social structures remained largely intact [79]. However, hormonal contraceptives, such as chlormadinone acetate, were found to alter female sexual behaviours, reducing heterosexual interactions while having no impact on homosexual interactions [80]. In other social species, sterilisation has been successfully applied to control overpopulation and mitigate human–wildlife conflicts. In capybaras, tubal ligation and vasectomy have proven effective for managing populations in urban areas while preserving social hierarchies [81]. Similarly, in coatis, uterine tubal ligation via mini-laparotomy has been implemented with minimal invasiveness and high success rates [82]. While sterilisation offers a humane alternative to lethal population control, logistical challenges include the need for repeated interventions, potential alterations in social dynamics, and ethical considerations. Thus, integrating sterilisation with other management strategies may provide a balanced approach to mitigating conflicts between humans and macaques.

The first key aspect of macaque management involves regulating population numbers and their geographical distribution. Effective management requires clear objectives, such as reducing harmful groups, preventing crop damage, or minimising disturbances to human living environments. Depending on the situation, the strategy may involve selective capture, partial capture, or total capture, but only after non-lethal regulatory measures have been attempted.

Selective capture involves removing up to 10% of a group annually, focusing on specific individuals that pose a threat or cause significant damage.

Partial capture is implemented for groups with a nuisance level of 7 or higher.

Total capture is reserved for groups with an appearance frequency level of 10 and a nuisance level of 8 or higher.

When carrying out captures, it is important to maintain a balanced group composition in terms of sex ratio and age structure, ensuring the group’s stability. Additionally, the most aggressive individuals should be targeted based on thorough observations.

To manage the geographical distribution of macaque groups, ecological continuity must be preserved to prevent excessive habitat fragmentation. Efforts should also account for the populations in neighbouring regions to avoid unintended impacts across administrative boundaries. A department’s management plan must ensure that actions do not significantly disrupt the overall distribution or connectivity of macaque populations.

Maintaining genetic diversity is critical for the long-term survival and stability of local populations. To achieve this, departments typically avoid relocating groups or individuals to distant locations, as this can disrupt genetic flow. Decisions regarding culling also take genetic diversity into account to ensure its preservation. For example, lethal measures are considered only if they are deemed necessary to prevent detrimental effects on the population’s genetic health (Kunio Watanabe, personal communication, 2023).

By carefully balancing population regulation, habitat continuity, and genetic diversity, macaque management strategies aim to address both human concerns and the long-term sustainability of macaque populations.

The second key aspect of managing Japanese macaques involves implementing targeted damage prevention measures tailored to the nuisance level of each group.

The third aspect focuses on managing the macaques’ natural environment, emphasising the conservation and restoration of forests and habitats. Preserving diverse natural vegetation is critical to providing sufficient food resources for macaques, thereby reducing their need to encroach on human settlements. Forest vegetation assessments are essential, and conservation and regeneration efforts are already underway to enable macaques to thrive in their natural habitats.

Edge management plays a vital role in reducing macaque incursions, as edge areas offer abundant food and shelter [20,83,84]. For interior forests, converting cypress plantations to native forests is often recommended to improve habitat quality [85,86]. A land-sharing strategy, which incorporates approaches like retention forestry, seeks to balance forestry activities with biodiversity conservation [87,88]. This approach is supported by research indicating that such practices can harmonise forestry and macaque conservation.

Approximately 42% of Japan’s forests are planted forests, most of which were established between 1950 and 1980 to meet post-war timber demands. However, many of these forests have not been adequately managed and are now reaching their planned harvest age. This lack of management has contributed to the decline of early-successional species, referred to as Japan’s ‘second crisis of biodiversity’. As a major timber importer, Japan has relied on overseas forests rather than utilising its own, raising discussions about reviving domestic plantation forestry. Restoration of young forests could provide suitable habitats for macaques and other species, particularly since only 30% of current planted forests will be needed to meet future timber demands. The remaining 70% could be restored to natural forests, either through harvesting or natural regeneration.

Retaining broad-leaved trees within conifer plantations has proven effective in enhancing habitat quality for macaques [84]. Long-rotation plantations (80–100 years) can improve habitat conditions to levels comparable to natural forests, while cypress trees, commonly used in Japanese forestry, serve as vital shelter from cold temperatures [84].

Discussions on adapting land-sharing and land-reserve strategies for habitat management are still in their early stages [23]. It is essential to view forests as habitats for diverse species and to integrate these strategies into a broader framework for local biodiversity conservation. Spatial statistical techniques now enable precise estimation of habitat units for various organisms, including macaques [21]. These methods help evaluate the impact of different forest management scenarios on habitat conservation and facilitate consensus among stakeholders.

By combining forest conservation with sustainable land-use strategies, it is possible to ensure the coexistence of humans and macaques while supporting broader biodiversity goals.

## 5. Discussion

Numerous prevention and control strategies are employed to address conflicts arising from interactions between Japanese macaques and human populations. These include the installation of high-quality fencing to protect sensitive areas, such as agricultural fields, and the removal of monkey attractants, such as unsecured food sources. Sophisticated warning systems are also used to anticipate and prevent potential damage to crops and property. However, human–macaque conflict management in Japan extends beyond physical control measures and is deeply influenced by cultural and religious factors [89,90,91]. Research perspectives on this issue are multidimensional and should include several key areas:

- Global Change Impacts: In-depth analysis of how global environmental changes affect macaque populations and their interactions with humans. It is linked to One Health concept.
- Non-Lethal Management Techniques: Development and evaluation of humane methods to mitigate conflicts. These techniques are critical as they address both biodiversity conservation [92,93] and societal concerns [94,95].
- Community-Based Practices: Investigating the effectiveness of community-led management practices and measures to restore macaques’ fear of humans.
- Cultural Dimensions: Understanding how variations in religion, perceptions of nature, and cultural practices influence human–macaque conflicts and management strategies [7,96].

Integrating the One Health [97] and One Conservation [98] frameworks into the management of human–macaque interactions offers a holistic approach that considers the interconnections between human, animal, and environmental health. One Health emphasises the importance of disease surveillance, reproductive health, and ecosystem conservation in primates, recognising that changes in reproductive strategies, habitat disruption, and disease dynamics directly impact both wildlife and human populations [99]. For instance, reproductive health studies in primates can inform conservation strategies by identifying species at risk due to low reproductive rates, aiding in both in situ and ex situ population management. Additionally, disease transmission risks between humans and macaques highlight the need for integrated surveillance and health interventions, particularly in urban and agricultural settings where macaques frequently interact with humans. One Conservation extends this perspective by emphasising the integration of in situ and ex situ conservation efforts, linking habitat restoration, genetic exchange, and sustainable management of both captive and wild populations [98]. This approach ensures that conservation strategies are not limited to protected areas but incorporate human-dominated landscapes and collaborative efforts across sectors, including agriculture and urban planning. The case of howler monkeys further illustrates the need for One Conservation, as their susceptibility to zoonotic diseases underscores the necessity of coordinated conservation and health efforts to protect both wildlife and human communities [100]. Applying these frameworks to Japanese macaque management would allow for more sustainable coexistence strategies, integrating ecological restoration, public health, and ethical considerations into conservation policies. By adopting these holistic approaches, human–macaque interactions can be managed more effectively, ensuring both species’ welfare while mitigating conflicts and promoting biodiversity conservation.

Integrating the cultural dimension is particularly important. Local beliefs and values, shaped by religion and views of nature, play a significant role in shaping how communities perceive and address these conflicts [101,102,103]. Exploring this dimension in greater depth is essential to devising strategies that foster harmonious coexistence between humans and macaques.

Further insights can be gained through adaptive management, international comparisons, and interdisciplinary approaches, which can lead to more sustainable solutions for this complex issue. However, it is also critical to reflect on the terminology used in these discussions. As Philippe Descola [104,105] notes, the term ‘management’ stems from a ‘naturalist’ framework, reflecting a modern dichotomy between nature and culture that often instrumentalises the former. Charles Stépanoff [106] describes the ‘paradoxical relationships between hunting, protection, and compassion,’ highlighting the tensions in such approaches.

This naturalist perspective contrasts with a more animist worldview, which may be better suited to the Japanese context. An animist approach, emphasising interconnectedness and mutual respect between humans and animals could provide a framework for rethinking conflict resolution strategies, fostering coexistence in ways that resonate with local cultural and spiritual values. Exploring these perspectives is crucial to achieving a holistic and sustainable approach to human–macaque coexistence.

## 6. Conclusions

The effective management of human–macaque conflicts in Japan requires a multi-faceted approach that integrates ecological, social, and ethical considerations. This review highlights the historical and contemporary challenges posed by macaque expansion into human-dominated landscapes, emphasising the need for adaptive and evidence-based strategies. While deterrence methods, habitat modifications, and fencing remain critical tools, the role of sterilisation and other fertility control techniques should be further explored as non-lethal alternatives. The One Health and One Conservation framework provides a holistic perspective, linking macaque management to broader issues of environmental sustainability, disease prevention, and biodiversity conservation. By incorporating these integrative approaches, alongside community engagement and scientific advancements, a more sustainable and ethical coexistence between humans and macaques can be achieved [107,108]. Future research should focus on refining non-lethal control measures, assessing their long-term effectiveness, and expanding interdisciplinary collaborations to ensure that management strategies remain adaptive to changing ecological and social conditions.

## Figures and Tables

**Table 1:** Determination grid for the levels set by the Japanese Ministry of the Environment.

**Points**	**Frequency of Appearance**	**Average Size During Appearance**	**Reaction Toward Humans**
0	Not visible (in deep forest)	The herd does not leave the mountain	Flee at the sight of humans even from a distance
1	Seen seasonally	Appearances of 2 to 3 individuals	Flee when people approach even from a distance
2	Regularly observed throughout the year (about once a week)	Appearances of fewer than 10 individuals	Do not flee when far away but do not approach within 20 m
3	Regularly observed throughout the year (two to three times a week)	Appearances of about 10 to 20 individuals	Some monkeys in the group do not flee when approached within 20 m
4	Observed throughout the year (almost daily)	Appearances of more than 20 individuals	Some monkeys in the group do not flee even when chased or approach people and threaten them
**Points**	**Situation of Damage to Villages**		**Damage to Daily Life**
0	No village or location affected by damage		No damage reported
1	Some villages suffer minor damage		Seen around residential areas
2	Some villages suffer significant damage		Enter gardens and climb onto roofs
3	Some villages suffer considerable damage		Damage objects/materials
4	At least three villages suffer considerable damage		Intrusions into homes have become common

**Table 2:** Nuisance level grid of the Japanese Ministry of the Environment.

Nuisance Level	Total Points
0	0
1	1–2
2	3–7
3	8–12
4	13–17
5	18–20

**Table 3:** Control and prevention strategies based on the nuisance level in Shiga Prefecture.

Nuisance Level	Prevention Strategies
1 and 2	—Nuisance elimination method: eliminate the causes attracting monkeys to villages (fruit trees, unharvested crops, organic waste).
	—Law on environmental modification of villages and agricultural lands: create physical or psychological barriers to make villages and agricultural areas less accessible to monkeys (agricultural roads, irrigation canals, buffer zones).
	—Deterrence methods.
3 and 4	—Nuisance elimination method.
	—Law on environmental modification of villages and agricultural lands.
	—Deterrence methods.
	—Proximity alert system: system used to detect the presence of monkeys and other wildlife and warn farmers and residents (motion sensors, surveillance cameras, sound, or visual alarms).
	—Intrusion prevention fencing: fencing only around agricultural lands using materials such as fishing nets, electric fences, or metal mesh greenhouses
5 and 6	—Nuisance elimination method.
	—Law on environmental modification of villages and agricultural lands.
	—Proximity alert system.
	—Scaring method: use of fireworks, firecrackers, firearms, or dogs to chase monkeys away.
	—Pursuit method: use of movement techniques, loud noises, and deterrent devices to scare animals and drive them to more appropriate areas.
	—Intrusion prevention fencing.
7 and more	—Nuisance elimination method: eliminate the causes attracting monkeys to villages (fruit trees, unharvested crops, and organic waste).
	—Law on environmental modification of villages and agricultural lands: create physical or psychological barriers to make villages and agricultural areas less accessible to monkeys (agricultural roads, irrigation canals, and buffer zones).
	—Deterrence methods.
	—Pursuit method.
	—Intrusion prevention fencing.
	—Habitat separation fencing: physically separate activity zones from human zones using posts, electric wires, or foundations.
	—Selective or total capture of a target group.

## Data Availability

Not applicable, as no new data were created or analysed in this study. Data sharing is not relevant to this article.
